# A Study to Assess the Feasibility of Utilising Virtual Reality for the Treatment of Accommodative and Vergence Infacility

**DOI:** 10.22599/bioj.175

**Published:** 2021-08-13

**Authors:** Alvin Munsamy, Husna Paruk

**Affiliations:** 1 University of KwaZulu-Natal, ZA

**Keywords:** accommodation, vergence, infacilities, virtual reality

## Abstract

**Background:**

To assess the feasibility, as well as viability, of utilising gaming using virtual reality (VR) to treat accommodative and vergence infacilities.

**Methods:**

Forty-two emmetropic and asymptomatic participants between the ages of 18 and 30, with normal binocular visual function, were selected for the study in 2018. Participants with binocular accommodative infacilities and/or vergence infacilities comprised the study population. The binocular accommodative facilities (BAF) were assessed using amplitude-scaled facilities (probe lens = 30% amplitude of accommodation; test distance = 45% amplitude of accommodation). All those with less than 10 cycles per minute (cpm) were regarded as failing. Vergence facilities were assessed using 12 pd base out and 3 pd base in prisms. All those with less than 15 cpm were regarded as failing. The participants were separated into age-matched experimental and control groups. The experimental group played a fast-paced game using Samsung Gear VR (SM-R323), whilst the control group watched a television film projected onto a two-dimensional screen at a distance of one metre. Pre-test and post-test binocular amplitude-scaled facilities and vergence facilities were measured for both groups after exposure for 25 minutes.

**Results:**

There was a significant, mean increase in binocular accommodative facilities of 4.67 ± 5.05 cpm (p = 0.008) for the experimental group (n = 12). There was a significant mean increase in vergence facilities of 3.72 ± 3.18 cpm (p < 0.001) for the experimental group (n = 32). A statistically significant mean difference of 4.07 cpm (95%CI: 0.97, 9.19; p = 0.03) between the respective control and experimental groups was found for binocular accommodative facilities and 2.45 cpm (95%CI: 0.68, 4.22; p = 0.008) for vergence facilities.

**Conclusion:**

Binocular accommodative facilities and vergence facilities increased after 25 minutes of VR gaming in asymptomatic emmetropic participants with accommodative infacilities and vergence infacilities. However, due to the small-scale, unmasked and unrandomised nature of the study more research is needed to confirm the results of this study.

## Introduction

The use of electronic devices in the activities of daily life is well established. They have been incorporated in vision therapy as an alternative to traditional vision therapy in order to encourage compliance and patient satisfaction. The appeal of virtual reality (VR) is increasing globally, spanning numerous sectors such as education, aviation, medicine and tourism. It is attractive as it engages the user in an alternate reality and provides an immersive experience.

Accommodative and vergence facilities are a measure of the stamina and dynamics of the vergence and accommodative response (Scheiman & Wick 2014). Patients who suffer from accommodative and vergence infacility may benefit from using VR by exploring the use of an alternative medium to traditional Hart charts and flippers, which may be daunting and require mental effort; and orthoptic treatment which is as effective if compliance is good (Hung, Ciuffreda & Semmlow 1986; Cooper et al. 1987; Sterner, Abrahamsson & Sjostrom 1999; Scheiman et al. 2005). The medium of VR has been used successfully with dichotic therapy in improving amblyopia and suppression (Aderman et al. 2015; Žiak et al. 2017). Turnbull et al. (Turnbull & Phillips 2017) assessed changes in binocular posture by assessing distance and near heterophoria, amplitude of accommodation and stereopsis in a VR simulation indoors, and VR-outdoors, in comparison to the real-world equivalent environments and found no changes after 40 minutes of exposure. The sample (mean age = 24.7 years) comprised nine emmetropes and 10 myopes with best correct acuity of 6/7.5. The change in amplitude of accommodation was –0.54D (p = 0.834) for VR-indoors when compared to outdoors and the real world. The change in stereopsis was –2.5 sec of arc (p = 0.533) for VR-indoors when compared to outdoors and the real world. However, the VERVE (Virtual Eye Rotation Vision Exercise) pilot study used VR to treat convergence insufficiency in a sample of nine symptomatic participants (mean age = 22.6 years), showed improvements in near-point of convergence (npc) (before = 10.4 cm; after = 5.3 cm; p = 0.005), positive fusional vergence (before = 14.7 pd; after = 25.1 pd; p = 0.03) and convergence insufficiency symptom survey (CISS) scores (before = 35.6; after = 19.9; p = 0.001) (Yaramothu et al. 2019).

Investigating the possibility of utilising e-devices as a medium to treat accommodative and vergence infacilities, with an appealing activity such as gaming, may encourage treatment. Patients may find therapy a laborious task and this may affect compliance. Access to games through mobile phone technology is becoming a favourite past-time. Virtual reality is used in entertainment for an immersive experience and appeals to consumers. The prospect of merging these two modalities as a therapy option for treatable vision options may encourage compliance, which may translate into functional cures. The judicious use of this medium must be distinguished from the gaming fraternity, who spend hours-on-end gaming for sport and income. A recent study warned that visual strain from continuous gaming may result in exophoria after 30 minutes (Mohamed Elias, Batumalai & Azmi 2019). Therefore, the formulation of future research must consider duration of exposure whilst balancing treatment exposure.

In a previous publication, we showed that, in an exploratory study, binocular accommodative and vergence facilities improved after VR gaming, irrespective of whether participants had an infacility disorder (Munsamy et al. 2020). This study included 42 participants who had passed or failed BAF and/or VF and showed a mean change of 2.24 cpm (p < 0.001) in binocular accommodative facility (BAF) and 3.81 cpm (p < 0.001) for vergence facility (VF) after 25 minutes of playing a game in a head-mounted VR device. This suggested its potential in treating both binocular accommodative and vergence infacilities, by studying only those who had failed pre-test BAF and/or VF. Therefore, the aim of the study was to investigate the use of VR gaming to assess its feasibility to treat patients with binocular accommodative and/or vergence infacility.

## Materials and Methods

The study was conducted at the University of KwaZulu-Natal, Westville Campus, in the Discipline of Optometry eye clinic. This feasibility study, conducted in 2018, used a quasi-experimental design that utilised non-probability purposive sampling to access participants, between the ages of 18 and 30 years, who failed binocular accommodative and vergence facility testing. The study consisted of two arms: an experimental group with accommodative infacilities and/or vergence infacilities which was exposed to playing a game in a VR device; and a control group with accommodative and/or vergence infacilities which was exposed to a fast-paced film on a flat screen. Secondary data from our previous exploratory study was utilised to conduct this study (Munsamy et al. 2020). The data for all participants from both experimental and control groups who failed binocular accommodative and/or vergence facilities were extracted to fulfil the present study’s aim.

### Selection criteria

Participants whose visual acuities were 6/6 at distance and 1M at near; refractive error of less than 0.50D with an inter-pupillary-distance between 55 mm and 70 mm (as per VR device); and a near point of convergence of less than 10 cm for satisfactory fusion to use the device. Spectacle and contact lens users, and individuals with a history of ocular and/or systemic disease and strabismus and heterophoric disorders were excluded from the study. Participants who failed accommodative and/or vergence facility testing (displayed less than 10 cpm for accommodative facility and less than 15 cpm for vergence facility) were included.

### Facility Measurements

*Binocular accommodative facilities* (BAF) were measured using amplitude-scaled facilities. This was chosen over traditional flippers of ±2D as the probe lens and the test distance is customised for the patient’s binocular amplitude of accommodation. In this way all participants are assessed based on their ability to stimulate and relax binocular accommodation. The probe lenses were determined using 30% of the individual participant’s binocular push-up amplitude of accommodation. The test distance was calculated using 45% of the participant’s binocular push-up amplitude of accommodation (Yothers, Wick & Morse 2002).

All push-up amplitudes of accommodation were measured using a RAF rule. The average of three binocular measurements of the first sustained blur was recorded. For illustrative purposes, if the amplitude was 20D the probe lens was ±3D and the test distance was set at 11 cm. This was standardised using a reference table (Scheiman & Wick 2014).

Participants were instructed to focus on a 20/30 target on an accommodative rock card which was held at the calculated test distance. The probe lenses were inserted into a flipper holder. Upon interposition of the first pair of probe lens, participants were asked to focus on a word until the letters become visible, at which point they informed the researcher. This was followed by interposing the second pair of probe lenses until the participant reported clarity. This was repeated for one minute using a stopwatch and a clicker to measure the number of cycles per minute (cpm). Binocular amplitude-scaled facilities were regarded as normal at greater than or equal to 10 cpm (Scheiman & Wick 2014).

*Vergence facility* (VF) was measured using 12 ^Δ^BO (base out) and 3 ^Δ^BI (base in) flipper prisms with participants focusing on a vertical row of 20/30 letters at a standard test distance of 40cm until fusion was achieved (Scheiman & Wick 2014). At first the 12 ^Δ^BO prism was introduced in front of one eye. This induced target diplopia, and the participant was then instructed to report binocular fusion when then the target became clear. Thereafter the 3 ^Δ^BI prism was introduced in front of one eye and the same procedure was followed as described above. When the target was fused and clear, one cycle was recorded. This was repeated for one minute using a stopwatch and a clicker to measure the number cycles per minute (cpm). The normal vergence facility using 12^Δ^BO/3^Δ^BI prisms was defined as greater than or equal to 15cpm (Scheiman & Wick 2014).

### Data Collection

A Samsung Gear VR (SM-R323) head-mounted device (HMD), powered by Oculus Rift, was utilised to provide the VR exposure. The field-of-view of the device was 101 degrees with convex lenses in the head-mounted device (HMD), with an inter-pupillary distance of 62mm. Data collection consisted of pre- and post-test binocular accommodative and vergence facility measurements. Participants played a fast-paced game on a Samsung smartphone using the VR headset.

The length of exposure to VR was informed by the preliminary findings before the commencement of data collection, which informed the appropriate gaming time required to elicit a change in accommodative and vergence facilities, for the minimum of 25 minutes of VR exposure. Pre-test BAF and VF measurements were obtained before the experimental group played a game in the VR-HMD; and the control group watched a fast-paced film on a non-VR computer screen (a two-dimensional projection screen).

After exposure, post-test accommodative and vergence facility measurements were obtained for each group. Vergence facility testing was alternated with accommodative facility testing for each participant during data acquisition, in order to prevent each testing procedure influencing the measurements. Examiners were unmasked, whilst participants were masked.

### Data analysis

Data were coded and entered into Microsoft Excel and exported to the Statistical Package for the Social Sciences (SPSS) version 24 for cleaning and analysis. Quantitative data were presented as means and standard deviations. The Kolmogorov-Smirnov test was used to test for the normality of data. Thereafter, the paired sample T-test was used to compare changes in the experimental and control groups, individually, after VR gaming and watching a film on a two-dimensional screen, respectively. Thereafter, the independent two-sample T-test was used to compare the changes noted in the experimental and control groups. The assumption of equality of variance of the independent sample T-test was checked using Levene’s test. All statistical tests were performed using two-sided tests, with the significance level set to α = 0.05.

## Results

### Binocular Accommodative Infacilities after VR gaming

**Figure 1:**
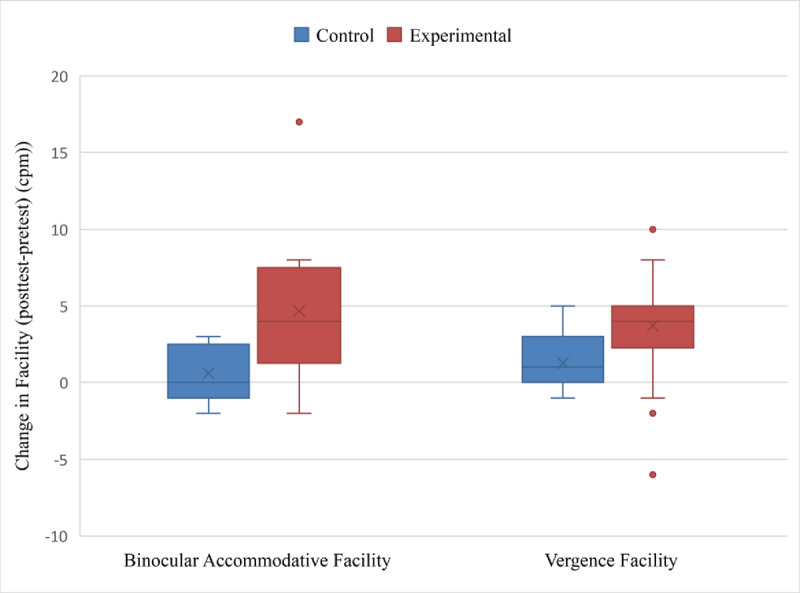
A box and whisker plot showing the change in facilities for the control and experimental groups after 25 minutes of gaming in VR device.

The experimental group with binocular accommodative infacilities compromised 12 participants, with a mean age of 21.42 ± 2.1 years, and with seven females and five males. The control group who had binocular accommodative infacilities consisted of five participants with a mean age of 21.20 ± 2.39 years, with one female and four males. The experimental group had mean pre-test facilities of 6.33 ± 2.22 cpm, and after VR gaming exhibited mean post-test facilities of 11.00 ± 4.88 cpm (***Table 1*** and ***Figure 1***).

**Table 1:** The effect of gaming using a VR device on participants with accommodative and vergence infacility.


FACILITY	GROUP	N	PRE-TEST MEAN ± SD (CPM)	POST-TEST MEAN ± SD (CPM)	MEAN CHANGE ± SD	95% CI (CPM)	P-VALUE

**BINOCULAR ACCOMMODATIVE FACILITY**	**Experimental**	12	6.33 ± 2.23	11.00 ± 4.88	+4.67 ± 5.05	1.45;7.88	0.008*

	**Control**	5	7.00 ± 1.73	7.60 ± 3.51	+0.60 ± 1.95	–1.82;3.02	0.529*

**VERGENCE FACILITY**	**Experimental**	32	9.84 ± 2.80	13.56 ± 4.02	+3.72 ± 3.18	n/a^##^	<0.001^#^

	**Control**	15	8.20 ± 2.51	9.47 ± 2.92	+1.27 ± 1.71	n/a^##^	0.015^#^


*Mean change*: Post-test, pre-test; (+) implies increase; (-) implies decrease; * Paired T-test; ^#^ Wilcoxon Signed Ranks test; ^##^ not applicable due to the use of non-parametric test.

Participants from the experimental group showed a statistically significant improvement of 4.67 ± 5.05 cpm (p = 0.008), whilst there were no statistically significant changes observed in the control group (***Table 1***). There was a statistically significant mean difference of 4.07 cpm (p = 0.01) between the experimental and control groups, which resulted from an improvement in the experimental group (***Table 2***).

**Table 2:** The comparison of mean changes in participants with accommodative and vergence infacility after 25 minutes of gaming using a VR device.


VARIABLE	GROUP	N	MEAN DIFFERENCE (CPM)	95% CI (CPM)	INDEPENDENT T-TEST

**BINOCULAR ACCOMMODATIVE FACILITY**	**Experimental**	12	+4.07	0.97; 9.1	0.03*

	**Control**	5			

**VERGENCE FACILITY**	**Experimental**	32	+2.45	0.68; 4.22	0.008

	**Control**	15			


*Mean difference*: Difference of means of experimental and control groups; (+): implies increase; *: equal variance not assumed.

### Vergence Infacilities after VR gaming

The experimental group comprised 32 participants with a mean age of 21.31 ± 2.1 years, with 17 females and 15 males. The control group consisted of 15 participants with a mean age of 20.00 ± 0.85 years, with 10 females and five males. The experimental group had a mean pre-test facility of 9.84 ± 2.80 cpm, and after VR gaming exhibited a mean post-test facility of 13.56 ± 4.02 cpm (***Table 1***).

Participants from the experimental group had a statistically significant improvement of 3.72 ± 3.18 cpm. There was also a statistically significant improvement of vergence facilities of 1.27 ± 1.71 cpm for the control group (***Table 1***). There was a statistically significant mean difference of 2.45 cpm (p = 0.008) between the experimental and control groups, which resulted from an improvement in the experimental group (***Table 2***).

## Discussion

Asymptomatic participants with binocular accommodative and vergence infacilities exposed to VR gaming for 25 minutes showed a statistically significant increase of BAF and VF. The improvement occurred after a single exposure, with BAF improving by approximately 4 cpm and VF improving by approximately 3 cpm. This shows that gaming using a VR device may be a viable option in the treatment of both binocular accommodative and vergence infacility. We do, however, remain circumspect, as these findings come from a study that is only at the feasibility stage.

VR, compared to real-world viewing, has de-coupling of accommodation and vergence. Although the headset is fixed for 3m, the headset is still closer; so accommodation and vergence may occur on different planes, creating a dissociation of accommodation and vergence, unlike natural viewing conditions where these are synchronised. Most recently, Elias et al. showed that playing a game in a VR environment cannot be compared to other VR tasks. It was found there was an increase in accommodative response of 0.22D in a young sample of 34 participants after 30 minutes of playing a game (Mohamed Elias, Batumalai & Azmi 2019). However, the study focused on visual strain; but the accommodation finding suggests increased stamina, which may help explain the increase in BAF in our study.

Turnbull et al. found no effect on binocular vision, and although our study showed an increase in facility, this may be in partial agreement, as neither study showed deterioration (Turnbull and Phillips 2017). Turnbull assessed changes in binocular posture of heterophoria; gaze stability; amplitude of accommodation and stereopsis in a VR headset and found no significant effects after 40 minutes of exposure. Kooi et al. showed that assessing AF in an HMD may be a satisfactory measurement of visual strain when using VR-HMD (Kooi 1997). Kang et al. then used this as a basis for assessing visual fatigue when using VR in an HMD. Kang et al. used an HMD-VR device to assess visual strain in 20 participants using binocular vision variables of NPC and AF and a visual fatigue survey after 20 minutes (Kang, et al. 2017). There was a significant increase in the symptom survey scores, indicating visual fatigue. However, the AF and NPC changes did not deteriorate to abnormal clinical measurements. The BAF reduced by 0.9 cpm (p = 0.306), whilst the near-point of convergence (NPC) reduced by 0.98 cm (p = 0.029). Although these studies were assessing visual strain, the clinical variable of AF allows for comparison with our study, which may disagree with Turnbull et al. —statistically but not clinically.

Other studies have shown that the accommodative-convergence conflict proved beneficial in treating amblyopia, suppression and convergence insufficiency (Aderman et al. 2015; Žiak et al. 2017; Yaramothu et al. 2019). The VERVE therapy pilot study also showed significant improvement in convergence insufficiency and the convergence insufficiency symptom survey after twelve, one-hour sessions (Yaramothu et al. 2019). Improvement was attributed to changes in the angular convergence demand which was created by the software. All these are binocular interventions that similarly show complementary improvements in various other areas of binocular disorders. All used the medium of VR in a gaming format that was also used in our study.

The improvement in the BAF experimental group in our study indicates a pass (greater than 10 cpm) and suggests possible treatment after a single exposure to VR. This was also true for the experimental group’s VF pass rate (greater than 15 cpm). The improvements may be due to the vergence accommodation and accommodative-vergence capability of adaptation in a VR environment to the stereoscopic demand (Eadie, Gray & Carlin 2000). It is believed this may help inform further research to explore the plausibility of the improvement in binocular accommodative facilities and vergence facilities after 25 minutes of gaming using a VR device, as well as the lasting effects thereof. However, it is yet to be established whether these improvements are long-lasting, to prove that the treatments are stable.

Our findings show that VR exposure did not cause the stamina of the accommodation and vergence system to deteriorate after 25 minutes. Participants who failed pre-test vergence and/or binocular accommodative facility tests had a statistically significant improvement in post-test facility measurements; thus suggesting the possibility of future studies of a treatment trial for accommodative and vergence infacilities to evaluate the lasting effects in symptomatic patients.

One of the study’s limitations was the use of asymptomatic participants. Further research is needed with a larger sample size to ascertain if the same outcome is repeated. Data gatherers were not masked during data collection. Therefore, one cannot rule out any undue influence on outcomes. The virtual reality display in this study did not allow for inter-pupillary distance of less than 55 mm.

The short exposure time may encourage further studies to consider the effect of repeated exposure on the impact of facilities. Longer exposure may be necessary to determine if the improvement in facilities noted in the study remains stable. Research using a randomised, controlled treatment trial with symptomatic and asymptomatic patients with vergence and/or accommodative infacilities, with repeated exposures over a period of time, is vital in determining a therapy regimen. It is therefore recommended that future studies follow up on participants a day, a week, a month and three months later, to ensure that the findings are lasting, to inform widespread clinical application. Further recommendations include ametropes using spectacles and contact lenses to establish if a similar outcome is achieved. It might also be useful to take a number of facility readings pre- and post-VR and use the means of these to determine if there is significant improvement after 25 minutes of VR. The inclusion of a paediatric population who suffer from either accommodative or vergence infacilities will be extremely valuable, as this may be a vulnerable population as the prevalence of infacilities is significant.

## Conclusion

The key findings of this study showed that accommodative and vergence facilities improved in asymptomatic participants who had binocular accommodative infacilities and vergence infacilities after exposure to gaming in a virtual reality headset for 25 minutes. These findings suggest an alternative treatment option for people who suffer from binocular accommodative infacilities and/or vergence infacilities. However, in the absence of a large-scale study on symptomatic participants, widespread public utilisation cannot be recommended. It is therefore proposed that a pilot study should be considered, consisting of a treatment trial on symptomatic patients with accommodative and/or vergence infacilities using a head-mounted VR-device. We do not recommend widespread clinical use until this is completed.

## Data accessibility statement

The datasets generated and/or analysed during the current study are not publicly available due to individual privacy but are available from the corresponding author on reasonable request.
